# Examining the impact of generative AI on student motivation and engagement: the mediating role of autonomy-support and autonomous motivation in education

**DOI:** 10.3389/fpsyg.2026.1852265

**Published:** 2026-06-30

**Authors:** Aldraiweesh Ahmed, Almutairy Sultan

**Affiliations:** Educational Technology Department, College of Education, King Saud University, Riyadh, Saudi Arabia

**Keywords:** artificial intelligence, educational technologies, Self-Determination Theory, SEM student motivation, student engagement

## Abstract

The rapid adoption of Generative Artificial Intelligence (GenAI) in higher education has transformed learning experiences; however, limited research has examined how psychological and motivational factors influence student engagement in AI-supported learning environments. Drawing upon Self-Determination Theory (SDT), Expectancy-Value Theory (EVT), and the Technology Acceptance Model (TAM), this study investigates the relationships among perceived autonomy, competence, relatedness, expectancy, value, autonomy support for AI use, autonomous motivation for AI use, student motivation, and student engagement. A quantitative research design was employed, and data were collected from 297 undergraduate and postgraduate students at King Saud University, Saudi Arabia. The proposed model was analyzed using Partial Least Squares Structural Equation Modeling (PLS-SEM). The findings revealed that perceived autonomy, perceived relatedness, and perceived value significantly enhanced autonomy support for AI use, while perceived autonomy, competence, and relatedness positively influenced autonomous motivation for AI use. Furthermore, autonomy support and autonomous motivation significantly increased student motivation, which subsequently emerged as the strongest predictor of student engagement. In contrast, perceived expectancy showed no significant influence on either autonomy support or autonomous motivation, while perceived competence did not significantly affect autonomy support. This study extends existing AI-in-education literature by integrating SDT, EVT, and TAM within a unified framework to explain student engagement in Generative AI-supported learning environments. Practically, the study provides valuable guidance for educators, instructional designers, and policymakers seeking to implement Generative AI technologies in ways that enhance meaningful learning experiences and sustainable student engagement in higher education.

## 1 Introduction

In recent years, the integration of artificial intelligence (AI) into education has received increasing scholarly attention because of its potential to transform teaching, learning, assessment, and student support practices (Huang and Tan, 2023). Among emerging AI technologies, Generative Artificial Intelligence (GenAI) has become especially influential in higher education because it can generate text, images, explanations, feedback, learning materials, and personalized academic support (Aithal and Aithal, 2023; Srinivasa et al., 2022). Since the public emergence of large language model-based tools such as ChatGPT, GenAI has rapidly reshaped how students search for information, complete assignments, receive feedback, and engage with learning content v (Batista et al., 2024; Chugh and Jain, 2026; Alwakid and Dahri, 2025a). These tools offer new opportunities for personalized learning, self-paced study, inclusive participation, academic writing support, and real-time feedback, which may enhance students’ motivation and engagement (Srinivasa et al., 2022; Yilmaz and Yilmaz, 2023).

Existing studies suggest that AI-supported learning environments can improve students’ academic experiences by offering adaptive feedback, intelligent tutoring, data-driven learning analytics, and interactive learning materials (Chugh and Jain, 2026; Alwakid and Dahri, 2025a). GenAI can further extend these benefits by enabling students to generate explanations, explore alternative viewpoints, ask follow-up questions, and receive immediate learning support (Ala et al., 2025; Alwakid et al., 2025). For postgraduate, international, and linguistically diverse students, GenAI may also reduce learning barriers by supporting language development, academic communication, and independent learning (Adekoya et al., 2026; Al-Rahmi et al., 2026; Dahri et al., 2024d). However, despite these advantages, recent literature also highlights serious concerns, including over-reliance on AI, reduced independent thinking, academic integrity issues, privacy concerns, assessment validity problems, and unequal access to AI technologies (Abubakar et al., 2025).

Student motivation and engagement remain central issues in higher education because they strongly influence learning persistence, academic performance, participation, and long-term educational success (Prentzas and Sidiropoulou, 2023). In AI-supported learning environments, motivation is not shaped only by the availability of technology but also by students’ psychological needs, perceived learning value, expectations of success, and the degree to which AI tools support autonomy and meaningful participation. Self-Determination Theory (SDT) explains that students become more intrinsically motivated when their basic psychological needs for autonomy, competence, and relatedness are satisfied (Ryan and Deci, 2020). In the context of GenAI, autonomy may be supported through flexible learning paths and student choice; competence may be enhanced through feedback and skill development; and relatedness may be strengthened through collaborative and inclusive learning opportunities (Dahri et al., 2024d; Alwakid and Dahri, 2025b).

Although SDT offers a strong psychological foundation, GenAI adoption in education also depends on students’ perceived expectancy and value. Expectancy-Value Theory (EVT) suggests that students are more likely to engage in learning activities when they believe they can succeed and when they perceive the task as useful, meaningful, or valuable (Ryan and Deci, 2017; Niemiec et al., 2009). In GenAI-supported learning, students’ perceived value of AI tools may influence whether they use them for deeper learning or only for task completion (Pasupuleti et al., 2025). Similarly, technology acceptance perspectives, particularly the Technology Acceptance Model (TAM), emphasize that students’ perceptions of usefulness, ease of use, and performance benefits influence their willingness to adopt educational technologies (Yuce et al., 2019; Bilquise et al., 2023). Recent studies increasingly argue that single-theory models are insufficient to explain GenAI adoption because student engagement is shaped by both psychological motivation and technology acceptance factors (Tbaishat et al., 2026).

Despite the growing body of research on GenAI in higher education, several important gaps remain. First, many studies focus mainly on AI adoption, perceived usefulness, or academic performance, while fewer examine the psychological mechanisms through which GenAI influences student motivation and engagement. Second, existing studies often examine SDT, EVT, or TAM separately, leaving limited understanding of how autonomy, competence, relatedness, expectancy, value, autonomy support, and autonomous motivation operate together in one integrated framework. Third, the mediating roles of autonomy support and autonomous motivation remain underexplored, particularly in relation to how students’ psychological perceptions are translated into motivation and engagement. Fourth, most prior studies rely heavily on Western or technologically advanced contexts, while empirical evidence from Saudi higher education remains comparatively limited (Chugh and Jain, 2026).

To address these gaps, this study examines how perceived autonomy, perceived competence, perceived relatedness, perceived expectancy, and perceived value influence autonomy support for AI use, autonomous motivation for AI use, student motivation, and student engagement. The study integrates SDT, EVT, and TAM to provide a more comprehensive explanation of GenAI-supported learning. By testing this integrated model using data from university students in Saudi Arabia, the study contributes to AI-in-education research by clarifying the motivational and psychological pathways through which GenAI may enhance or limit student engagement.

Accordingly, this study addresses the following research objectives:

1. To examine the effects of perceived autonomy, competence, relatedness, expectancy, and value on autonomy support and autonomous motivation in GenAI-supported learning.
2. To investigate how autonomy support and autonomous motivation influence student motivation and engagement.
3. To test an integrated SDT–EVT–TAM-based model for explaining student engagement in AI-supported higher education.
4. To provide theoretical and practical implications for responsible and motivation-supportive GenAI integration in higher education.

Based on these objectives, the study addresses the following research questions:

1. How do students’ perceived autonomy, competence, relatedness, expectancy, and value influence autonomy support and autonomous motivation in GenAI-supported learning?
2. How do autonomy support and autonomous motivation affect student motivation and engagement?
3. How can an integrated SDT–EVT–TAM framework explain student engagement in GenAI-supported higher education?

## 2 Theoretical background

Understanding the impact of generative artificial intelligence (AI) on student motivation and engagement necessitates detailed exploration of various motivational theories. This study integrates several key theoretical frameworks to examine how AI influences the learning experience and outcomes.

Self-Determination Theory (SDT), developed by Deci and Ryan, is fundamental to analyzing motivational processes in educational settings (Zhou and Li, 2023). SDT asserts that human motivation is driven by the fulfillment of three basic psychological needs: autonomy, competence, and social relatedness. Autonomy refers to the need to feel in control of one’s actions and decisions (Deci et al., 1994). Competence pertains to the desire to feel effective and capable in one’s activities, and relatedness involves the need for meaningful connections with others (Ryan and Deci, 2020; Ryan and Deci, 2008). Research based on SDT highlights that environments that support these needs enhance intrinsic motivation, engagement, and overall well-being among learners (Deci et al., 1994). For instance, in educational contexts, when students perceive that their learning environment supports their autonomy, helps them feel competent, and fosters meaningful relationships, they are more likely to engage deeply and be intrinsically motivated (Deci et al., 1994).

Generative AI can play a significant role in promoting autonomy-supportive learning environments by offering personalized learning experiences (Jeno et al., 2023). For example, AI-powered adaptive learning systems adjust instructional content based on individual students’ progress and preferences, allowing for a more self-directed learning approach (Almogren et al., 2024; Soomro et al., 2024). This adaptation aligns with students’ autonomy by enabling them to navigate their learning paths and make choices that reflect their personal goals and interests (Yilmaz and Yilmaz, 2023; Reeve, 2009). Moreover, AI facilitates creative problem-solving and collaborative projects, enhancing students’ sense of ownership over their learning experiences and further supporting their sense of autonomy (Makri et al., 2023).

Expectancy-Value Theory (EVT), developed by Eccles and Wigfield, complements SDT by focusing on how the perceived value of tasks influences students’ motivation. According to EVT, motivation is determined by the perceived value of a task and the expectancy of success (Ryan and Deci, 2017; Niemiec et al., 2009). It identifies dimensions of value, such as intrinsic, attainment, and utility values (Vashishth et al., 2024). Intrinsic value relates to the inherent enjoyment derived from the activity, attainment value concerns the importance of doing well for one’s self-identity, and utility value refers to the perceived usefulness of the activity for future goals (Wigfield and Eccles, 2000). For instance, students might be motivated to engage with an AI-powered learning tool if they find it enjoyable, if doing well with it is important for their self-identity, or if it provides benefits for their future academic or career goals (Wigfield and Eccles, 2000).

Generative AI has the potential to increase students’ sense of value in their learning activities by making content more interactive and engaging. AI applications can design simulations, virtual laboratories, and gamified learning environments that fit the interests of students as well as their future career goals, thus maximizing both the intrinsic and utility value of the activities (Jeno et al., 2023). By linking learning activities with individual student goals and interests, AI has tremendous potential to increase motivation and engagement (Malinka et al., 2023).

The Technology Acceptance Model (TAM) also contributes to the enrichment of the understanding of the impact of AI by covering technology adoption (Davis, 1989). TAM stresses that perceived ease of use and perceived usefulness are essential determinants of technology acceptance (Yuce et al., 2019; Bilquise et al., 2023). In TAM, learners are more likely to use AI tools if they find them useful and worthwhile in respect to their learning goals. This explains why students’ belief in the value and usefulness of AI tools has the potential to affect their motivation and commitment (Venkatesh and Davis, 2000). When students identify the value and simplicity of AI tools, they tend to embrace and effectively incorporate them into their learning activities.

Integrating generative AI into learning follows these theoretical approaches by designing adaptive learning platforms that respond to students’ motivational drivers and psychological needs (Tao et al., 2022; Vaughan, 2014; Dahri et al., 2024b; Dahri et al., 2021). AI applications that provide personalized feedback, instant support, and interactive material can help students develop a sense of autonomy, improve their competence, and establish social connections (Yuce et al., 2019; Bilquise et al., 2023; Dahri et al., 2023a). For instance, artificially intelligent chatbots and virtual tutors can interact with students in dialogue-based learning activities, offering personalized feedback and scaffolding assistance in real time, which in turn maintains students’ engagement and motivation.

By integrating theories such as SDT, EVT, and TAM, this study aims to provide a comprehensive understanding of how generative AI influences student motivation and engagement (Azevedo et al., 2022). These theoretical perspectives provide valuable insights into how AI can be utilized to enhance educational practices, leading to more effective and learner-centered interventions (Luo et al., 2021).

## 3 Proposed research model and hypothesis development

Generative Artificial Intelligence (AI) technology shows great potential to revolutionize how learners learn and how teachers teach in the dynamic area of education. A well-established theory in educational psychology, “Self-Determination Theory (SDT),” is at the nucleus of the proposed theoretical model (Ryan and Deci, 2020; Ryan and Deci, 2017). SDT focuses on the significance of meeting psychological needs and promoting intrinsic motivation. According to this theory, people are motivated to engage in behaviors that fulfill their basic psychological needs for” relatedness, competence, and autonomy” (Ryan and Deci, 2017). Within the context of education and generative AI, SDT posits that autonomous-supportive AI systems, characterized by adaptive feedback mechanisms and individualized learning trajectories, increase students’ perceived control over their learning processes, leading to increased motivation and engagement (Madjar and Assor, 2013). Along with SDT, the proposed model integrates Expectancy-Value Theory (EVT), which sheds light on how task expectations and task value impact motivation (Şahin et al., 2024).

Originally formulated by Wigfield and Eccles (2000), EVT is concerned with how people’s beliefs about succeeding and the perceived value they give to tasks influence their behavior and motivation (Eccles and Wigfield, 2023). According to EVT, students’ motivation is greatly determined by their expectations of succeeding at a task and the value they give to the outcomes of the task. More particularly, EVT argues that when students find a task valuable and useful, and they are confident that they will be able to accomplish it, their motivation to accomplish the task increases (Wigfield and Eccles, 2000; Eccles and Wigfield, 2024).

Research supports the integration of EVT into the model by highlighting that perceived utility and task value are critical in shaping students’ willingness to engage with AI-assisted learning activities. For instance, Eccles and Wigfield (2023) found that students’ perceptions of the usefulness and relevance of learning tools directly impacted their engagement and persistence. Moreover, the model incorporates the TAM, which underscores the role of AI use in influencing behavior. Also posits that students’ perceptions of the AI system’s feedback accuracy and its ability to facilitate social interactions— that is, perceived relatedness—positively influence both the autonomy support and autonomous motivation derived from the AI system (Jeno et al., 2023).

By integrating SDT, EVT, and TAM, the proposed model aims to provide a comprehensive framework for understanding how generative AI technologies can enhance educational outcomes through nuanced consideration of psychological needs, task value, and social interactions.

**Figure 1:**
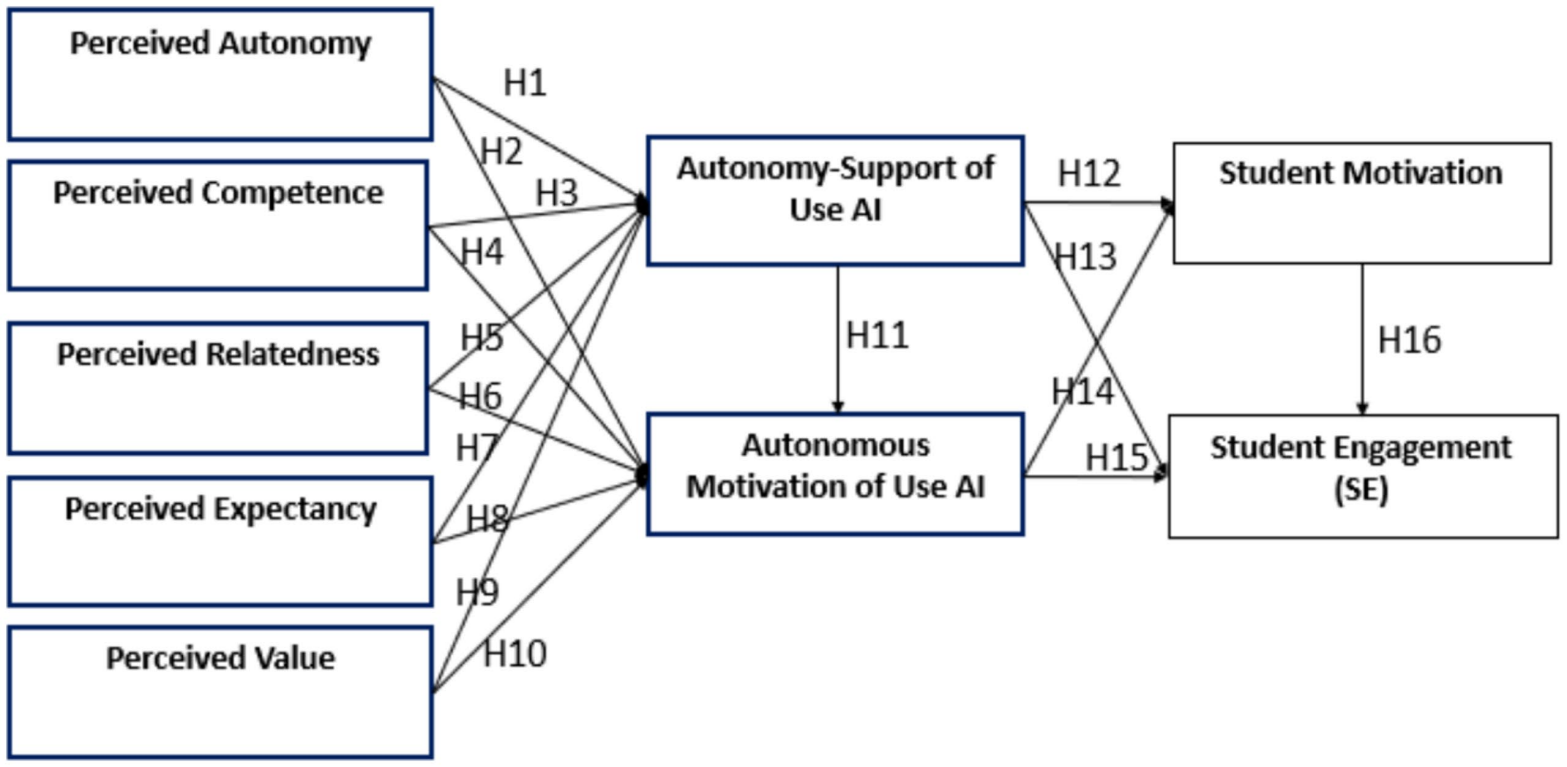
Propose research model.

Figure 1 shows the proposed theoretical model, in which several hypotheses are formulated to elucidate the relationships among the key constructs. Accordingly, the following constructs and hypotheses are defined.

### 3.1 Perceived autonomy

Perceived autonomy refers to individuals’ perception of having control over their actions and decisions within a given context. In the educational context, perceived autonomy is crucial for fostering intrinsic motivation and engagement (Ryan and Deci, 2008; Vansteenkiste et al., 2006). Previous research has demonstrated that autonomy-supportive environments enhance students’ perceived autonomy, leading to greater motivation and academic success (Madjar and Assor, 2013).

AI systems designed to support autonomy often feature adaptive feedback and personalized learning paths that align with the principles of autonomy support (Dahri et al., 2024a). These features are intended to enhance students’ sense of control over their learning processes. Previous research indicates that when educational tools, including AI systems, offer students choices and control, they experience higher levels of autonomy support (Lazorak et al., 2021). Studies have shown that autonomy-supportive environments in AI applications lead to more positive user experiences and improved learning outcomes (van der Vorst and Jelicic, 2019). Therefore, we hypothesized that higher perceived autonomy would be associated with increased autonomy support provided by AI systems.

*H1:* Higher perceived autonomy leads to increased autonomy support in AI use.

Autonomous motivation, which refers to engaging in activities out of intrinsic interest rather than external pressure, is significantly influenced by perceived autonomy (Parrisius, 2020; Manning, 2012). When students feel that they have control over their learning, their intrinsic motivation to engage with AI tools increases (Dahri et al., 2024d; Almogren et al., 2024). Research supports the notion that autonomy-enhancing features in AI-driven educational tools lead to higher levels of autonomous motivation, as students are more likely to engage meaningfully with the technology (Alamri et al., 2020). Consequently, we hypothesized that a higher sense of autonomy would lead to greater autonomous motivation for using AI for educational purposes.

*H2:* Higher perceived autonomy leads to greater autonomous motivation for AI use.

### 3.2 Perceived competence

Perceived competence reflects individuals’ beliefs about their ability to perform tasks and achieve desired outcomes effectively. Perceived competence is vital in shaping students’ motivation, persistence, and academic achievement (Ryan and Deci, 2020; Ryan and Deci, 2017). Studies have shown that interventions to enhance perceived competence improve student engagement and learning outcomes (Vansteenkiste et al., 2006).

AI systems that cater to students’ perceived competence often include features that provide constructive feedback and adjust difficulty levels to match users’ abilities (Hooda et al., 2022). Research has shown that students who believe they are competent are more likely to perceive AI tools as supportive and effective (Dahri et al., 2024c; Dahri et al., 2023b). This increased perception of competence can lead to greater autonomy support from AI systems, as these tools are designed to adapt and provide personalized learning experiences. For instance, AI tools that adjust their feedback based on students’ abilities help maintain an optimal challenge level, which aligns with competence-supportive features (Alamri et al., 2020; Hooda et al., 2022). Therefore, we hypothesized that higher perceived competence is associated with increased autonomy support provided by AI systems.

*H3:* Higher perceived competence results in increased autonomy support in AI use.

As soon as students sense themselves as competent to utilize AI tools, their autonomous motivation to work with these tools is apt to increase. This is because perceived competence fuels feelings of achievement and efficacy, which are vital for promoting autonomous motivation (Chiu et al., 2023; Xia et al., 2022). Research shows that AI tools that support user competence are likely to be associated with higher levels of motivation and commitment (Jo, 2023). As students gain confidence and mastery through AI-aided activities, their autonomous motivation to use technology continues to develop. Therefore, we hypothesized that greater perceived competence would result in more autonomous motivation for AI use.

*H4:* Higher perceived competence leads to greater autonomous motivation in AI use.

### 3.3 Perceived relatedness

Perceived relatedness is defined as the perception of being connected to, belonging to, and having social support in one’s learning environment. In AI-assisted education, perceived relatedness includes students’ feelings about how well the AI system can offer constructive feedback, enable social interaction, and foster a supportive learning community. The literature suggests that building perceived relatedness increases students’ motivation, engagement, and well-being (Ryan and Deci, 2020; Ryan and Deci, 2017).

AI systems that encourage perceived relatedness are usually programmed to allow social interaction and provide personalized feedback that fosters a sense of belonging. The findings show that when learners are supported and feel connected to their learning software, the tools feel more supportive of their autonomy (Jo, 2023). Social simulation or personalized support by AI systems makes learning more interactive and encouraging, which should make students perceive greater autonomy support (Ryan, 1991). Therefore, we can predict that greater perceived relatedness will lead to more autonomy support from AI systems.

*H5:* Higher perceived relatedness leads to increased autonomy support in AI use.

Perceived relatedness significantly impacts autonomous motivation. When students feel a sense of connection and support, their intrinsic motivation to engage with AI tools increases (Jo, 2023; Ryan, 1991). AI systems that foster a supportive and interactive learning environment can enhance students’ sense of belonging and engagement, leading to greater motivation to use these tools autonomously (Xia et al., 2022). As students experience increased relatedness through meaningful interactions and feedback, their motivation to utilize AI tools independently and effectively is likely to increase. Therefore, we hypothesized that a higher perceived relatedness would lead to greater autonomous motivation for AI use.

*H6:* Higher perceived relatedness leads to greater autonomous motivation for AI use.

### 3.4 Perceived expectancy

Perceived expectancy refers to students’ beliefs about the effectiveness and usefulness of AI technologies in supporting their learning goals and outcomes (Tannady and Dewi, 2024). It encompasses students’ expectations regarding the AI system’s ability to provide accurate feedback, personalized recommendations, and adaptive learning experiences. Previous studies have highlighted the importance of perceived expectancy in shaping students’ acceptance and utilization of AI-driven educational tools (Davis et al., 1989).

When students have high expectations of the effectiveness of AI systems, they anticipate that these tools will significantly enhance their learning experience. AI systems that are perceived as highly effective in providing accurate and personalized support are likely to be seen as more autonomy supportive (Haerens et al., 2018). Research shows that when students believe in the capability of AI systems to meet their learning needs and provide valuable feedback, they perceive these systems as better supporting their autonomy (Lemos et al., 2017; Hu et al., 2021). Thus, we hypothesized that higher perceived expectancy would lead to increased autonomy support provided by AI systems.

*H7:* Higher perceived expectancy results in increased autonomy support in AI use.

Perceived expectancy also influences students’ intrinsic motivation. When students expect AI technologies to be effective in supporting their learning objectives, their motivation to engage with these tools autonomously is enhanced (Yurt and Kasarci, 2024). The anticipation of receiving valuable, personalized feedback and adaptive recommendations can significantly boost students’ intrinsic motivation to use AI tools (Hu et al., 2021). Consequently, we hypothesized that higher perceived expectancy would result in greater autonomous motivation for AI use.

*H8:* Higher perceived expectancy leads to greater autonomous motivation in AI use.

### 3.5 Perceived value

Perceived value refers to the benefits, relevance, and importance students attribute to AI-supported learning activities and resources (Petrick, 2002). It encompasses students’ evaluations of the potential gains and rewards of using AI technologies in education. Research suggests that enhancing perceived value increases motivation, engagement, and satisfaction with AI-driven learning experiences (Petrick, 2002).

When students perceive high value in AI-supported learning activities, they are more likely to view these technologies as providing meaningful and relevant support to their learning. This perception often translates into a greater sense of autonomy support from AI systems (Cui and van Esch, 2022). Research indicates that when students believe that AI technologies offer substantial benefits and relevance, they are more inclined to view these tools as supportive of their learning autonomy (Cui and van Esch, 2022; Lalicic and Weismayer, 2021). Thus, we hypothesized that a higher perceived value would result in increased autonomy support in AI use.

*H9:* Higher perceived value leads to increased autonomy support in AI use.

Perceived value also plays a significant role in shaping students’ intrinsic motivation. When students recognize the substantial benefits and relevance of AI tools, their motivation to engage with these technologies autonomously is likely to increase (Cui and van Esch, 2022). The perceived rewards and advantages of using AI in learning can enhance students’ intrinsic motivation to utilize these tools effectively (Cui and van Esch, 2022; Lalicic and Weismayer, 2021). Consequently, we hypothesized that a higher perceived value would lead to greater autonomous motivation for AI use.

*H10:* Higher perceived value leads to greater autonomous motivation for AI use.

### 3.6 Autonomy-support of use AI

Autonomy support of AI refers to the degree to which artificial intelligence systems in education facilitate students’ sense of autonomy by providing choice, control, and self-directed learning opportunities. Research suggests that autonomy-supportive AI systems can enhance students’ motivation and engagement by promoting feelings of competence and autonomy (Jeno et al., 2023).

Autonomy-supportive AI systems contribute to students’ intrinsic motivation by providing opportunities for self-direction and control over their learning processes. When AI tools are designed to support autonomy, they help students feel more competent and self-motivated in their learning. Prior studies have shown that such supportive environments enhance intrinsic motivation by aligning with students’ psychological needs for autonomy (Hooda et al., 2022). Therefore, we hypothesized that increased autonomy support from AI systems would lead to greater autonomous motivation among students.

*H11:* Autonomy support in AI use fosters greater autonomous motivation.

AI systems that offer autonomy support can positively impact overall student motivation. By enabling students to make choices and exercise control over their learning, these systems contribute to higher levels of motivation (Calvo et al., 2020). The literature supports this notion, demonstrating that autonomy-supportive features in educational technologies can enhance students’ general motivation by making learning experiences more engaging and personally relevant (Chiu et al., 2023). Thus, we hypothesized that autonomy support in AI use would have a positive effect on student motivation.

*H12:* Autonomy support in AI use positively influences student motivation.

Furthermore, autonomy-supportive AI systems are expected to enhance student engagement. Engaging with AI tools that support autonomy allows students to actively participate in their learning, leading to increased engagement (Chiu et al., 2023). Research indicates that when students perceive their learning environment as supporting their autonomy, they are more likely to be engaged and invested in their learning experiences (Chiu, 2021). Therefore, we hypothesized that autonomy support in AI use would positively influence student engagement.

*H13:* Autonomy support in AI use positively impacts student engagement.

### 3.7 Autonomous motivation of use AI

Autonomous motivation for AI use refers to the internal drive and interest students experience when engaging in AI-supported learning activities. Autonomous motivation is characterized by a sense of volition and self-determination, which is essential for sustaining long-term engagement and academic success (Ryan and Deci, 2020). Research suggests that promoting autonomous motivation in educational settings leads to more significant learning outcomes and well-being (Gunter and Reeves, 2017).

Students autonomously motivated to use AI tools are likely to experience increased overall motivation. This type of motivation, rooted in personal interest and volition, encourages deeper engagement with learning activities. Studies have demonstrated that when students are motivated by internal factors, such as personal interest or perceived relevance, their motivation to learn tends to improve significantly (Parrisius, 2020). Thus, we hypothesized that autonomous motivation for AI use would positively influence student motivation.

*H14:* Autonomous motivation in AI use positively influences student motivation.

Autonomous motivation plays a crucial role in enhancing student engagement. When students are intrinsically motivated to use AI tools, they are more likely to actively engage with their learning materials and processes (Hidayat-ur-Rehman, 2024). Research indicates that autonomous motivation fosters a higher level of involvement and persistence in learning activities, as students are driven by their personal interests and sense of choice (Wei, 2023); therefore, we hypothesized that autonomous motivation for AI use would have a positive effect on student engagement.

*H15:* Autonomous motivation in AI use positively impacts student engagement.

### 3.8 Student motivation

Student motivation drives students’ behaviors, choices, and actions in the learning process (Skinner et al., 2016; Ifinedo, 2017). It encompasses a complex interplay of intrinsic and extrinsic factors that influence students’ willingness to engage in learning activities and pursue academic goals. Previous research has identified various motivational theories, such as Self-Determination Theory (SDT) and Expectancy-Value Theory, to understand and enhance student motivation in educational settings (Petrick, 2002; Sánchez-Fernández and Iniesta-Bonillo, 2007).

Motivated students are generally more engaged in learning activities. Engagement, characterized by active participation and sustained attention, is often a direct outcome of high motivation levels. Research has shown that when students are motivated, whether through intrinsic factors such as interest or extrinsic incentives, their engagement levels typically rise (Wei, 2023; Zepke, 2011). Consequently, we hypothesized that student motivation would positively influence student engagement, reflecting the connection between how motivated students are and their level of involvement in learning activities.

*H16:* Student motivation positively influenced student engagement.

### 3.9 Student engagement

Student engagement refers to the degree of active participation, involvement, and investment that students demonstrate in their learning experiences. It encompasses the cognitive, behavioral, and emotional dimensions of student learning and is considered a key predictor of academic success and learning outcomes (Lin and Huang, 2018). Research suggests that promoting autonomy, competence, and relatedness can foster greater student engagement in educational activities, leading to enhanced learning experiences and achievements (Lin and Huang, 2018; Pintrich, 2003) (see Figure 1).

## 4 Research methodology

### 4.1 Research design

This study adopted a quantitative, cross-sectional research design grounded in the positivist research paradigm. A quantitative approach is appropriate when examining causal relationships among latent constructs and testing theoretically derived hypotheses using statistical techniques. The study investigates the relationships among psychological factors, autonomy-supportive learning environments, autonomous motivation, student motivation, and student engagement within the context of Generative Artificial Intelligence (GenAI)-supported education.

The conceptual framework was developed by integrating Self-Determination Theory (SDT), Expectancy-Value Theory (EVT), and the Technology Acceptance Model (TAM). These theories collectively explain how students’ psychological needs, perceived value, expectancy beliefs, and technology-related perceptions influence motivation and engagement in educational settings. Since the proposed model contains multiple latent constructs and mediating relationships, Partial Least Squares Structural Equation Modeling (PLS-SEM) was selected as the primary analytical approach. PLS-SEM is particularly suitable for prediction-oriented studies, theory extension, and complex models involving multiple endogenous constructs and mediation effects (Hair and Alamer, 2022; Henseler et al., 2015).

### 4.2 Sample and data collection procedure

The study sample consisted of 297 undergraduate and postgraduate students enrolled at King Saud University, Saudi Arabia. Data collection was conducted during April 2024 using an online survey administered through institutional mailing lists and university-supported online platforms.

A convenience sampling strategy was employed because the study focused specifically on students with direct exposure to contemporary digital learning technologies and Generative AI tools. Convenience sampling is widely utilized in educational technology and behavioral research when investigating emerging technological phenomena within accessible populations (Etikan et al., 2016). Participation was voluntary, and respondents were informed about the objectives of the study before completing the survey. All participants provided informed consent electronically before accessing the questionnaire. To ensure data quality, incomplete responses and duplicate submissions were removed before analysis.

### 4.3 Instrument development procedure

Data were collected using a structured questionnaire developed from previously validated measurement scales reported in the educational psychology, technology acceptance, and AI adoption literature. To ensure content validity, all measurement items were adapted from established instruments and modified to reflect the context of Generative AI use in higher education. The survey instrument consisted of 38 items distributed across nine latent constructs: perceived autonomy, perceived competence, perceived relatedness, perceived expectancy, perceived value, autonomy-support of AI use, autonomous motivation of AI use, student motivation, and student engagement. All items were measured using a five-point Likert scale ranging from 1 (“strongly disagree”) to 5 (“strongly agree”). The use of previously validated scales improves construct validity and facilitates comparability with prior studies (Hair and Alamer, 2022). Minor wording modifications were made to align the items with the context of AI-supported learning while preserving the theoretical meaning of the original constructs. Table 1 presents the construct definitions, item sources, and number of measurement items used in this study.

**Table 1:** Survey tool.

Construct	No. of items	References
Perceived autonomy	5	Jeno et al. (2023)
Perceived competence	5	Jeno et al. (2023)
Perceived relatedness	4	Wood (2016)
Perceived expectancy	4	Tannady and Dewi (2024) and Prihadi et al. (2012)
Perceived value	4	Petrick (2002)
Autonomy-support of use AI	4	Jeno et al. (2023)
Autonomous motivation of use AI	4	Jeno et al. (2023)
Student motivation	4	Yilmaz and Yilmaz (2023) and Skinner et al. (2016)
Student engagement (SE)	4	Tao et al. (2022) and Skinner et al. (2016)

### 4.4 Pilot testing of the survey instrument

Before the main data collection phase, a pilot study involving 50 students from the same target population was conducted. The purpose of the pilot study was to assess questionnaire clarity, item relevance, wording appropriateness, and preliminary reliability. Participants in the pilot study were asked to provide feedback regarding question comprehension, ambiguity, and contextual relevance to Generative AI-supported learning. Based on the feedback received, several items were revised to improve readability and conceptual clarity. Internal consistency reliability was evaluated using Cronbach’s alpha. The results indicated that all constructs achieved Cronbach’s alpha values greater than 0.70, exceeding the recommended threshold for acceptable reliability (Hair and Alamer, 2022; Nunnally and Bernstein, 1994). These findings confirmed the reliability and suitability of the instrument for large-scale data collection.

### 4.5 Ethical considerations

This study adhered to established ethical standards for educational and behavioral research. Participation was entirely voluntary, and respondents were informed about the purpose of the study before completing the questionnaire. Participants were assured that their responses would remain anonymous and confidential and would be used solely for academic research purposes. No personally identifiable information was collected, and respondents were free to withdraw from participation at any stage without penalty. Electronic informed consent was obtained from all participants before commencing the survey. The study complied with institutional ethical guidelines for research involving human participants.

### 4.6 Common method bias assessment

As all data were collected using a self-reported survey instrument, common method bias (CMB) was assessed before hypothesis testing. Following Podsakoff et al. (2003), Harman’s single-factor test was performed by loading all measurement items into an exploratory factor analysis. The results indicated that the first factor accounted for less than 50% of the total variance, suggesting that common method bias was not a serious concern In addition, full collinearity variance inflation factors (VIFs) were examined following Kock (2016). All VIF values were below the recommended threshold of 3.3, indicating that common method bias and multicollinearity were unlikely to threaten the validity of the findings.

### 4.7 Data analysis procedure

The collected data were analyzed using SmartPLS 4 software. The analysis followed the two-stage procedure recommended by Hair and Alamer (2022), consisting of measurement model evaluation and structural model evaluation.

In the first stage, the measurement model was assessed by examining indicator loadings, Cronbach’s alpha, composite reliability (CR), average variance extracted (AVE), and discriminant validity. Convergent validity was confirmed when factor loadings exceeded 0.70, composite reliability exceeded 0.70, and AVE values exceeded 0.50.

Discriminant validity was evaluated using the Fornell–Larcker criterion and the Heterotrait-Monotrait Ratio (HTMT). HTMT values below 0.85 indicate satisfactory discriminant validity.

In the second stage, the structural model was assessed through path coefficient estimation, coefficient of determination (*R*^2^), predictive relevance (Q^2^), effect size (f^2^), and bootstrapping procedures. A bootstrapping procedure with 5,000 resamples was performed to determine the statistical significance of direct and indirect relationships among constructs.

Multicollinearity was examined using variance inflation factor (VIF) values, where values below 5.0 indicate the absence of problematic collinearity (Hair and Alamer, 2022). Predictive relevance was assessed using the blindfolding procedure, while effect sizes were evaluated using Cohen’s (1988) guidelines. This comprehensive analytical approach ensured robust assessment of both the measurement properties of the constructs and the hypothesized structural relationships within the proposed SDT–EVT–TAM framework.

## 5 Results

### 5.1 Participant profile analysis

Table 2 presents the demographic and AI-related characteristics of the respondents. Among the 297 participants, females represented 55.9% of the sample, while males accounted for 44.1%. Most respondents were between 18 and 22 years old (67.3%), indicating that the sample was predominantly composed of traditional university-age students. Undergraduate students constituted the majority of participants (85.2%), whereas postgraduate students represented 14.8% of the sample. Regarding academic specialization, Humanities Sciences accounted for the largest proportion (55.2%), followed by Scientific Sciences (23.9%) and Medical Sciences (20.9%).

**Table 2:** Demographic and AI usage characteristics of participants.

Variable	Category	Count	Percentage (%)
Gender	Female	166	55.9
	Male	131	44.1
Age (years)	18–22	200	67.3
	23–26	62	20.9
	27–30	12	4.0
	31–34	8	2.7
	More than 35	15	5.1
Academic level	Undergraduate	253	85.2
	Postgraduate	44	14.8
Specialization	Humanities Sciences	164	55.2
	Scientific Sciences	71	23.9
	Medical Sciences	62	20.9
Prior experience with generative AI	Beginner	87	29.3
	Intermediate	145	48.8
	Advanced	65	21.9
Frequency of AI use	Daily	168	56.6
	Weekly	96	32.3
	Monthly	33	11.1
Self-reported digital literacy	Basic	52	17.5
	Intermediate	154	51.9
	Advanced	91	30.6
Primary use of generative AI	Academic Writing	103	34.7
	Assignment Support	87	29.3
	Information Searching	64	21.5
	Coding/Programming	23	7.7
	Other Purposes	20	6.8

To provide a more comprehensive profile of AI adoption among participants, additional AI-related characteristics were examined. Nearly half of the respondents reported intermediate experience with Generative AI tools (48.8%), while 29.3% identified themselves as beginners and 21.9% reported advanced experience. The results further revealed that Generative AI technologies were frequently used among participants, with 56.6% indicating daily use and 32.3% reporting weekly use. Only a small proportion (11.1%) used AI tools every month.

Regarding digital literacy, most students perceived themselves as having intermediate (51.9%) or advanced (30.6%) digital skills, suggesting adequate technological readiness for AI-supported learning environments. Academic writing (34.7%) and assignment support (29.3%) emerged as the most common applications of Generative AI, followed by information searching (21.5%). These findings indicate that Generative AI has become an integral component of students’ academic activities and learning processes within higher education.

### 5.2 Measurement model

Convergent validity pertains to the degree of correlation between various measures that assess the same construct (Hair et al., 2010). We evaluated the convergent validity of the constructs in our study by analyzing the factor loadings, Cronbach’s alpha, composite reliability (CR), and average variance extracted (AVE), as presented in Table 3. These indications provide proof of the dependability and accuracy of the measurement approach (Dahri et al., 2024b; Hair et al., 2014; Al-Rahmi et al., 2021).

**Table 3:** Convergent validity.

Construct	Items	Factor loadings	Cronbach’s alpha	CR	AVE
Perceived autonomy	PA1	0.83	0.89	0.92	0.69
	PA2	0.88			
	PA3	0.88			
	PA4	0.82			
	PA5	0.73			
Perceived competence	PC1	0.7	0.82	0.88	0.59
	PC2	0.76			
	PC3	0.83			
	PC4	0.78			
	PC5	0.76			
Perceived relatedness	PR1	0.81	0.81	0.87	0.63
	PR2	0.85			
	PR3	0.71			
	PR4	0.8			
Perceived expectancy	PE1	0.77	0.74	0.83	0.56
	PE2	0.8			
	PE3	0.77			
	PE4	0.75			
Perceived value	PV1	0.74	0.82	0.88	0.65
	PV2	0.67			
	PV3	0.89			
	PV4	0.9			
Autonomy-support of use AI	ASU1	0.85	0.89	0.92	0.75
	ASU2	0.85			
	ASU3	0.88			
	ASU4	0.88			
Autonomous motivation of use AI	AMU1	0.8	0.77	0.85	0.59
	AMU2	0.67			
	AMU3	0.78			
	AMU4	0.82			
Student motivation	SM1	0.78	0.77	0.85	0.6
	SM2	0.77			
	SM3	0.85			
	SM4	0.85			
Student engagement (SE)	SE1	0.86	0.79	0.88	0.71
	SE2	0.9			
	SE3	0.75			

Factor loadings represent the strength of the relationship between each item and its corresponding construct (Hair et al., 2010). All factor loadings in our study exceeded the recommended threshold of 0.7 (Hair et al., 2010), indicating a strong convergent validity. For example, factor loadings for items measuring perceived autonomy ranged from 0.73 to 0.88, while those for perceived value ranged from 0.67 to 0.90.

#### 5.2.1 Cronbach’s alpha

Cronbach’s alpha assesses the internal consistency reliability of the items within each construct (Fornell and Larcker, 1981; Otman et al., 2025). All constructs demonstrated high internal consistency, with Cronbach’s alpha values exceeding 0.70, the recommended threshold (Hair et al., 2010). For instance, perceived autonomy had a Cronbach’s alpha of 0.89, perceived competence had a value of 0.82, and autonomy-support of using AI was 0.89 (Hair et al., 2014; Raffaghelli et al., 2012).

#### 5.2.2 Composite reliability

CR measures the extent to which the items in a construct are consistent in their reflection of that construct (Raffaghelli et al., 2012; Yang et al., 2025). Similar to Cronbach’s alpha, all constructs exhibited high composite reliability, surpassing the threshold of 0.70 (Hair et al., 2010). For instance, autonomy-support of use AI had a CR of 0.92, perceived value had a CR of 0.88, and student engagement had a CR of 0.88 (Hair et al., 2010; Hair et al., 2019).

#### 5.2.3 Average variance extracted

The AVE reflects the proportion of variance captured by the construct with the variance due to measurement error. AVE values above 0.50 are considered acceptable (Hair et al., 2010). In our study, all constructs achieved AVE values above this threshold, indicating adequate convergent validity. For example, perceived autonomy had an AVE of 0.69, perceived relatedness had an AVE of 0.63, and student motivation had an AVE of 0.60 (Hair et al., 2010). Significant factor loadings, Cronbach’s alpha values, CR, and AVE all indicated that the measurement model had great convergent validity. These findings support the validity and reliability of the constructs assessed in our study (Table 3).

Discriminant Validity is defined as the degree to which one model construct differs from another is determined by discriminant validity. The Heterotrait-Monotrait (HTMT) ratio and Fornell-Larcker criterion were used to assess discriminant validity (Henseler et al., 2015). HTMT Ratio: The HTMT ratio compares the correlations between constructs to determine whether they are significantly lower than 1. A value below 0.85 indicates discriminant validity (Henseler et al., 2015). In our study, Table 4 shows that all HTMT ratios were below this threshold, indicating adequate discriminant validity. For example, the highest HTMT ratio was 0.85 between autonomous motivation (AMU) and student motivation (SM), suggesting that these constructs are sufficiently distinct (Henseler et al., 2015).

**Table 4:** Discriminant validity (HTMT ratio).

Construct	AMU	ASU	PA	PC	PE	PR	PV	SE	SM
AMU									
ASU	0.85								
PA	0.76	0.75							
PC	0.72	0.64	0.69						
PE	0.66	0.63	0.53	0.73					
PR	0.76	0.76	0.64	0.69	0.77				
PV	0.77	0.82	0.75	0.75	0.82	0.82			
SE	0.77	0.69	0.71	0.6	0.55	0.57	0.72		
SM	0.85	0.74	0.74	0.61	0.62	0.6	0.78	1.03	

According to the Fornell–Larcker criterion, the square root of each construct’s Average Variance Extracted (AVE) should be greater than its correlations with other constructs (Fornell and Larcker, 1981). Table 5 presents these results. The diagonal values (square roots of AVEs) are all higher than the corresponding off-diagonal correlation values, confirming that each construct shares more variance with its indicators than with other constructs. For example, the square root of the AVE for AMU (0.77) is greater than its correlations with ASU (0.72), PA (0.64), and other constructs. Similarly, ASU (0.86), PA (0.83), SE (0.84), and the remaining constructs all met this requirement, demonstrating that discriminant validity was established, indicating that the constructs were empirically distinct from one another. Furthermore, the HTMT ratio values (reported separately) were all below the conservative threshold of 0.85, further supporting the discriminant validity. Hence, both the Fornell–Larcker criterion and the HTMT ratio provide evidence that the measurement model adequately distinguishes between constructs, ensuring that each represents a unique theoretical concept.

**Table 5:** Discriminant validity (Fornell–Larcker criterion).

Construct	AMU	ASU	PA	PC	PE	PR	PV	SE	SM
AMU	0.77								
ASU	0.72	0.86							
PA	0.64	0.67	0.83						
PC	0.58	0.55	0.59	0.77					
PE	0.54	0.56	0.46	0.59	0.75				
PR	0.61	0.65	0.54	0.56	0.64	0.8			
PV	0.66	0.73	0.67	0.62	0.65	0.68	0.8		
SE	0.61	0.58	0.59	0.48	0.44	0.45	0.59	0.84	
SM	0.67	0.62	0.62	0.49	0.49	0.47	0.62	0.83	0.77

### 5.3 Structural model analysis

The coefficient of determination (*R*^2^) indicates the proportion of variance in the endogenous (dependent) constructs explained by the exogenous (independent) constructs in the model (Hair et al., 2010; Hair et al., 2019). In our study, we calculated the R-squared values for each construct to assess their explanatory power. The *R*^2^ values ranged from 0 to 1, with higher values reflecting greater explanatory power. Generally, values of 0.75, 0.50, and 0.25 are considered substantial, moderate, and weak, respectively (Hair et al., 2019).

As shown in Table 6, the *R*^2^ values for autonomous motivation (AMU) and autonomy-support of AI use (ASU) are 0.610 and 0.620, respectively. This suggests that 61 and 62% of the variance in AMU and ASU, respectively, is explained by their predictors, representing a moderate-to-substantial level of explanatory power. The *R*^2^ value for student engagement (SE) was 0.700, indicating that the independent variables accounted for 70% of the variance in SE, which can be considered substantial. Meanwhile, the *R*^2^ for student motivation (SM) was 0.490, showing that the predictors explained 49% of the variance in SM, which was at a moderate level. The adjusted *R*^2^ values were slightly lower than the unadjusted values, as expected, because they correct for the number of predictors in the model. Nevertheless, the *R*^2^ values collectively demonstrate that the proposed model explains a meaningful and substantial amount of variance across the dependent constructs, confirming its explanatory strength.

**Table 6:** Model fitness score.

Construct	R-square	R-square adjusted
AMU	0.610	0.600
ASU	0.620	0.620
SE	0.700	0.690
SM	0.490	0.480

#### 5.3.1 Effect size (f^2^)

In addition to evaluating the explanatory power of the model through *R*^2^ values, the effect size (f^2^) was assessed to determine the contribution of each predictor construct to the endogenous variables. According to Cohen (1988), f^2^ values of 0.02, 0.15, and 0.35 indicate small, medium, and large effects, respectively. The results revealed that perceived autonomy, perceived relatedness, perceived value, autonomy-support of AI use, and autonomous motivation exerted moderate-to-large effects on their corresponding endogenous constructs. Conversely, perceived competence and perceived expectancy exhibited relatively weaker effects, which is consistent with their non-significant structural relationships reported in the hypothesis testing results. These observed effect sizes indicate that the proposed predictors contribute meaningfully to explaining variations in autonomy-support of AI use, autonomous motivation, student motivation, and student engagement. These findings further strengthen the explanatory capability of the proposed SDT–EVT–TAM framework.

#### 5.3.2 Predictive relevance (Q^2^)

The predictive relevance of the structural model was evaluated using the Stone–Geisser Q^2^ statistic obtained through the blindfolding procedure. All endogenous constructs achieved Q^2^ values greater than zero, indicating that the model possesses satisfactory predictive relevance. In particular, autonomous motivation, autonomy-support of AI use, student motivation, and student engagement demonstrated moderate-to-high predictive capability, confirming that the model can effectively predict the target constructs beyond the sample data. These findings suggest that the proposed framework has acceptable out-of-sample predictive performance and provides meaningful predictive insights regarding students’ motivational and engagement behaviors in Generative AI-supported learning environments.

#### 5.3.3 Collinearity assessment (VIF)

Prior to assessing the structural relationships, collinearity among the predictor constructs was examined using Variance Inflation Factor (VIF) values. The analysis indicated that all VIF values remained well below the recommended threshold of 5.0 and below the more conservative threshold of 3.3 suggested for assessing common method bias. These results confirm the absence of problematic multicollinearity among the predictor constructs and indicate that common method bias is unlikely to threaten the validity of the structural model estimates. Therefore, the path coefficients can be interpreted with confidence. The structural model demonstrated satisfactory explanatory power, meaningful effect sizes, adequate predictive relevance, and no evidence of problematic collinearity. These findings provide strong evidence supporting the robustness, predictive capability, and practical applicability of the proposed model in explaining student motivation and engagement in Generative AI-supported educational environments.

#### 5.3.4 Hypothesis testing results

**Figure 2:**
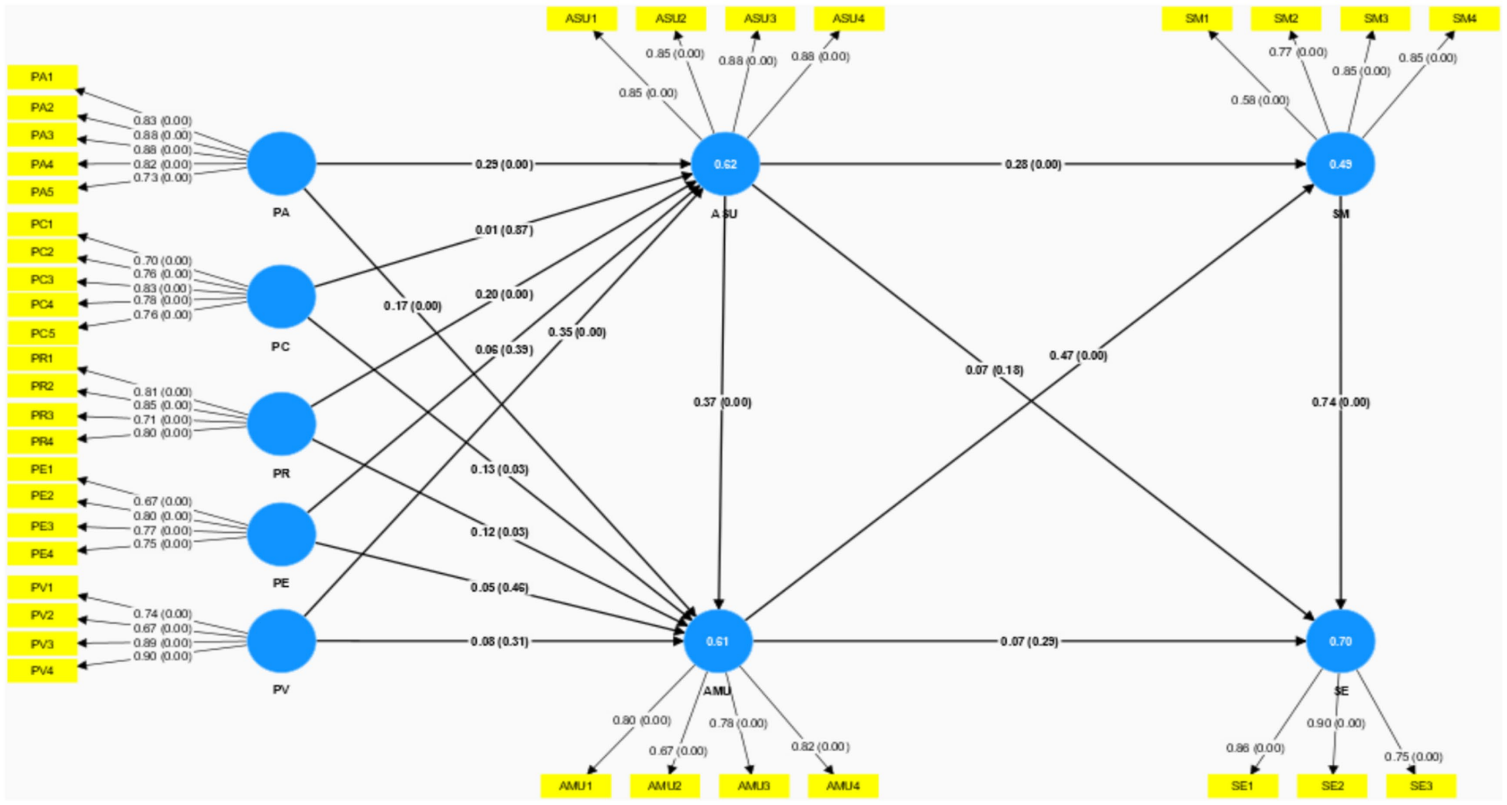
Structural model (path coefficients).

The results of the hypothesis testing are presented in Table 7 and Figure 2. Several hypothesized relationships were supported, while others were not, offering important insights into the drivers of autonomy support, autonomous motivation, and downstream outcomes in AI-enabled education. Perceived autonomy had a significant positive effect on both autonomy support for AI use (H1: *β* = 0.290, *T* = 4.840, *p* < 0.001) and autonomous motivation for AI use (H2: *β* = 0.170, *T* = 2.980, *p* < 0.01). These findings confirm that higher levels of perceived autonomy enhance both the perception of support for autonomy and individuals’ intrinsic motivation to use AI. Perceived competence also showed significant positive effects on autonomous motivation (H4: *β* = 0.130, *T* = 2.150, *p* < 0.05), although its effect on autonomy support (H3) was negligible and nonsignificant. Similarly, perceived relatedness significantly predicted both autonomy support (H5: *β* = 0.200, *T* = 3.150, *p* < 0.01) and autonomous motivation (H6: *β* = 0.120, *T* = 2.160, *p* < 0.05), highlighting the importance of social connectedness in driving motivation and support for the adoption of AI. Perceived value significantly influenced autonomy support (H9: *β* = 0.350, *T* = 4.470, *p* < 0.001), although its effect on autonomous motivation (H10) was not significant. Importantly, autonomy support for AI use strongly predicted autonomous motivation (H11: *β* = 0.370, *T* = 5.330, *p* < 0.001) and student motivation (H12: *β* = 0.280, *T* = 4.230, *p* < 0.001). Furthermore, autonomous motivation significantly enhanced student motivation (H14: *β* = 0.470, *T* = 7.250, *p* < 0.001), and student motivation strongly predicted student engagement (H16: *β* = 0.740, *T* = 14.010, *p* < 0.001), indicating that motivation is a critical pathway linking psychological needs and engagement.

**Table 7:** Hypothesis testing (path, *T*-value, and *P*-value).

Hypothesis	Path (*β*)	*T* statistics	*P* values	Results
H1 = Perceived Autonomy → Autonomy-Support of Use AI	0.290	4.840	0.000	Accepted
H2 = Perceived Autonomy → Autonomous Motivation of Use AI	0.170	2.980	0.000	Accepted
H3 = Perceived Competence → Autonomy-Support of Use AI	0.010	0.160	0.870	Rejected
H4 = Perceived Competence → Autonomous Motivation of Use AI	0.130	2.150	0.030	Accepted
H5 = Perceived Relatedness → Autonomy-Support of Use AI	0.200	3.150	0.000	Accepted
H6 = Perceived Relatedness → Autonomous Motivation of Use AI	0.120	2.160	0.030	Accepted
H7 = Perceived Expectancy → Autonomy-Support of Use AI	0.060	0.860	0.390	Rejected
H8 = Perceived Expectancy → Autonomous Motivation of Use AI	0.050	0.740	0.460	Rejected
H9 = Perceived Value → Autonomy-Support of Use AI	0.35	4.47	0.00	Accepted
H10 = Perceived Value → Autonomous Motivation of Use AI	0.080	1.020	0.310	Rejected
H11 = Autonomy-Support of Use AI → Autonomous Motivation of Use AI	0.370	5.330	0.000	Accepted
H12 = Autonomy-Support of Use AI → Student Motivation	0.280	4.230	0.000	Accepted
H13 = Autonomy-Support of Use AI → Student Engagement	0.070	1.350	0.180	Rejected
H14 = Autonomous Motivation of Use AI → Student Motivation	0.470	7.250	0.000	Accepted
H15 = Autonomous Motivation of Use AI → Student Engagement	0.070	1.060	0.290	Rejected
H16 = Student Motivation → Student Engagement	0.74	14.01	0.00	Accepted

The effects of perceived competence on autonomy support (H3: *β* = 0.010, *T* = 0.160, *p* > 0.05) and perceived expectancy on both autonomy support (H7: *β* = 0.060, *T* = 0.860, *p* > 0.05) and autonomous motivation (H8: *β* = 0.050, *T* = 0.740, *p* > 0.05) were not significant. Likewise, the influence of perceived value on autonomous motivation (H10: *β* = 0.080, *T* = 1.020, *p* > 0.05) was also rejected. Additionally, the direct effects of autonomy support on student engagement (H13: *β* = 0.070, *T* = 1.350, *p* > 0.05), and autonomous motivation on student engagement (H15: *β* = 0.070, *T* = 1.060, *p* > 0.05) were not significant.

These results highlight that perceived autonomy, relatedness, competence, and value are significant predictors of autonomy support and autonomous motivation in AI use (see relationships in Figure 2). Autonomy support and autonomous motivation, in turn, exert strong effects on student motivation, which is a key driver of student engagement. Notably, direct links from autonomy support and autonomous motivation to engagement were not supported, suggesting that the impact of these constructs on engagement is fully mediated by motivation.

## 6 Discussion

This study examined the impact of Generative Artificial Intelligence (GenAI) on student motivation and engagement by integrating Self-Determination Theory (SDT), Expectancy-Value Theory (EVT), and technology acceptance perspectives. The findings provide important insights into the psychological mechanisms through which students interact with AI-supported learning environments and contribute to the growing literature on Generative AI in higher education.

The results demonstrate that perceived autonomy significantly influences both autonomy-support of AI use and autonomous motivation for AI use. These findings support the fundamental assumptions of Self-Determination Theory, which proposes that autonomy is a core psychological need that enhances intrinsic motivation and engagement (Deci et al., 1994; Ryan and Deci, 2020). Students who perceived greater control and flexibility in their interactions with AI tools were more likely to experience autonomy-supportive learning environments and develop autonomous motivation toward AI-assisted learning. These findings are consistent with recent studies indicating that Generative AI promotes personalized learning, self-paced study, and learner agency, thereby strengthening students’ willingness to adopt and effectively utilize AI technologies (Adekoya et al., 2026; Yang et al., 2025; Shoukat, 2024).

Perceived competence demonstrated a significant positive effect on autonomous motivation but did not significantly influence autonomy-support of AI use. This finding partially supports SDT and suggests that students who feel capable of using AI technologies are more intrinsically motivated to engage with them. Recent evidence suggests that AI-driven feedback, adaptive tutoring, and personalized learning support can strengthen students’ competence perceptions and improve learning performance (Otman et al., 2025). However, competence alone may not be sufficient to create perceptions of autonomy support, which may depend more strongly on instructional practices and learning environment characteristics.

The findings further reveal that perceived relatedness positively influences both autonomy-support and autonomous motivation. These results reinforce the importance of social connectedness in AI-supported learning environments and are consistent with previous studies emphasizing the role of supportive relationships in fostering motivation and engagement (Ryan and Deci, 2008; Reeve, 2009; Vansteenkiste et al., 2006). Recent Generative AI research similarly suggests that AI-supported collaborative learning environments can enhance inclusivity, peer interaction, and social support, particularly among diverse student populations (Adekoya et al., 2026). Consequently, educational institutions should ensure that AI implementation complements rather than replaces meaningful social interactions.

Interestingly, perceived expectancy did not significantly influence either autonomy-support or autonomous motivation. This finding differs from several previous technology acceptance studies (Jeno et al., 2023; Azevedo et al., 2022; Tannady and Dewi, 2024). One possible explanation is that students have become increasingly familiar with AI technologies, reducing the importance of expectancy beliefs as a motivational driver. Recent studies suggest that perceived learning value and intrinsic motivational factors explain Generative AI adoption more effectively than expectancy beliefs alone (Bai and Yang, 2025). Furthermore, the rapid diffusion of AI tools may have normalized their use, making performance expectations less influential than psychological and contextual factors.

Perceived value was found to significantly influence autonomy-support of AI use but not autonomous motivation. This finding indicates that students who recognize the educational value of AI technologies are more likely to perceive supportive learning environments that encourage AI utilization. This result aligns with previous research highlighting perceived value as a critical determinant of technology adoption (Jeno et al., 2023; Vansteenkiste et al., 2006). Recent studies consistently identify perceived learning value as one of the strongest predictors of Generative AI adoption, continuance intention, and educational engagement (Ng and Naval, 2025). However, the absence of a direct effect on autonomous motivation suggests that perceived usefulness alone may not be sufficient to foster intrinsic motivation, which may require additional psychological and pedagogical support.

The results further demonstrate that autonomy-support of AI use significantly enhances both autonomous motivation and student motivation. These findings strongly support SDT and confirm that autonomy-supportive learning environments play a critical role in developing self-determined forms of motivation (Ryan and Deci, 2020; Ryan and Deci, 2017). Recent studies have similarly shown that instructor support, ethical AI guidance, and autonomy-supportive educational practices positively influence students’ continued use of AI tools and their motivation to engage in learning activities (Luo and Day, 2026). Therefore, educational institutions should focus on creating learning environments that empower students to use AI responsibly and independently.

However, autonomy-support of AI use did not directly influence student engagement. This finding suggests that autonomy support alone may not be sufficient to produce immediate engagement outcomes. Instead, its effects appear to operate indirectly through motivational mechanisms. Recent literature indicates that while autonomy-supportive environments facilitate AI adoption and continuance intention, engagement outcomes often depend on additional factors such as motivation, AI literacy, instructional design quality, and institutional support (Ode et al., 2025). This finding highlights the importance of considering multiple pathways through which AI-supported learning environments influence student behavior.

Similarly, autonomous motivation for AI use did not directly affect student engagement. Although autonomous motivation positively influenced student motivation, it did not translate directly into engagement behaviors. This result contrasts with some previous studies (Jeno et al., 2023; Reeve, 2009). but aligns with emerging research suggesting that engagement is influenced by a combination of motivational, contextual, and instructional factors rather than motivation alone. Recent studies indicate that learning strategies, disciplinary differences, teacher support, and the quality of AI-mediated learning experiences may moderate the relationship between motivation and engagement (Adekoya et al., 2026).

The strongest relationship observed in the model was between student motivation and student engagement (*β* = 0.74, *p* < 0.001). This finding confirms that motivation remains the primary mechanism driving students’ active participation in AI-supported learning activities. The result is consistent with previous educational research (Song and Song, 2023; Skinner and Belmont, 1993). and is further supported by recent Generative AI studies demonstrating that motivated learners exhibit higher levels of cognitive, emotional, and behavioral engagement when interacting with AI technologies (Dahri and Moskalenko, 2024; Dahri et al., 2025). Consequently, enhancing student motivation should remain a central objective of AI integration strategies in higher education. The findings support the integration of SDT, EVT, and technology acceptance perspectives in explaining student engagement within AI-supported learning environments. The results indicate that psychological needs, perceived educational value, and autonomy-supportive learning conditions play more important roles in fostering engagement than technology-related factors alone. These findings contribute to the emerging body of knowledge on Generative AI in higher education and provide empirical evidence supporting the development of human-centred, motivationally supportive, and ethically responsible AI-enhanced learning environments.

### 6.1 Theoretical implications

This study makes several important theoretical contributions to the growing body of literature on Generative Artificial Intelligence (GenAI) in higher education. First, the findings provide empirical support for Self-Determination Theory (SDT) by demonstrating that perceived autonomy and perceived relatedness significantly influence autonomy-support of AI use and autonomous motivation for AI use. These results reinforce the central proposition of SDT that satisfaction of basic psychological needs promotes self-determined forms of motivation, which subsequently enhance learning engagement and educational outcomes (Ryan and Deci, 2020). The significant effects of autonomy-support and autonomous motivation further confirm the relevance of SDT in explaining student behavior within AI-supported learning environments.

Second, this study extends Expectancy-Value Theory (EVT) by examining the roles of perceived expectancy and perceived value in the context of Generative AI-supported education. While perceived value exhibited significant positive effects on students’ motivational processes, perceived expectancy did not demonstrate a significant influence on autonomy-support or autonomous motivation. This finding suggests that in emerging AI-assisted learning environments, students may place greater emphasis on the perceived usefulness and educational value of AI tools than on their expectations of successful performance. Consequently, the study contributes to the evolving EVT literature by highlighting potential shifts in motivational drivers within technology-enhanced educational contexts.

Third, the study contributes to the Technology Acceptance Model (TAM) literature by demonstrating that technology-related perceptions alone may be insufficient to explain student engagement with Generative AI tools. Instead, the findings indicate that psychological factors and motivational mechanisms play critical mediating roles between students’ perceptions of AI and their engagement behaviors. This supports recent calls for integrating psychological and technology acceptance theories to better understand AI adoption and usage in higher education.

A major theoretical contribution of this research lies in the integration of SDT, EVT, and TAM into a unified framework. Previous studies have often examined these theories independently, resulting in fragmented explanations of AI adoption and educational outcomes. By simultaneously investigating psychological needs, motivational beliefs, and technology-related perceptions, this study provides a more comprehensive understanding of how Generative AI influences student motivation and engagement. The results suggest that autonomy-support and autonomous motivation serve as critical mechanisms through which students translate their perceptions of AI technologies into meaningful engagement with learning activities.

Furthermore, this study advances the emerging literature on Generative AI in education by moving beyond technology adoption and usage intention perspectives. While much of the existing research focuses on acceptance, perceived usefulness, or behavioral intention, the present study examines the motivational pathways that connect AI-supported learning experiences to student engagement. The findings therefore contribute to a deeper theoretical understanding of how Generative AI affects students’ psychological experiences and educational behaviors.

Finally, the study provides empirical evidence from the Saudi Arabian higher education context, which remains underrepresented in the current Generative AI literature. By validating the proposed model within a non-Western educational setting, the study contributes to the cross-cultural generalizability of motivational and technology acceptance theories and provides a foundation for future comparative investigations across diverse educational environments.

### 6.2 Practical implications

The findings of this study offer several practical implications for educators, policymakers, higher education institutions, and educational technology developers seeking to integrate Generative Artificial Intelligence (GenAI) effectively into teaching and learning processes. First, the significant effects of perceived autonomy and relatedness suggest that educators should design AI-supported learning environments that encourage student choice, self-directed learning, and meaningful interaction. Providing learners with flexibility in how they use AI tools can enhance their sense of ownership, motivation, and engagement.

Second, the positive influence of perceived value highlights the importance of demonstrating the educational benefits of GenAI. Universities and instructors should provide clear guidance on how AI tools can support learning, problem-solving, critical thinking, and academic productivity. Training workshops and AI literacy programs can help students understand the appropriate and effective use of AI technologies in educational contexts.

Third, the findings emphasize the critical role of autonomy-supportive practices in fostering autonomous motivation and student engagement. Educators should move beyond merely providing access to AI tools and instead create supportive learning environments that encourage exploration, feedback, reflection, and independent learning. Such practices can maximize the motivational benefits of GenAI while reducing the risks of over-reliance and passive learning.

Finally, policymakers and educational technology developers should establish clear ethical guidelines, responsible AI-use policies, and user-centered AI systems that align with students’ learning needs. By combining technological innovation with psychologically supportive educational practices, institutions can create more engaging, inclusive, and sustainable AI-enhanced learning environments that promote both academic success and student well-being.

## 7 Conclusion

This study aimed to examine the impact of generative AI on student motivation and engagement, focusing on the mediating roles of autonomy support and autonomous motivation within the framework of Self-Determination Theory (SDT). Using a sample of 297 undergraduate students from King Saud University in Saudi Arabia, the data were analyzed using Partial Least Squares Structural Equation Modeling (PLS-SEM). This methodological approach allowed for rigorous testing of the hypothesized relationships between key psychological constructs—perceived autonomy, competence, relatedness, expectancy, and value—and student outcomes in AI-enabled learning environments.

The hypothesis testing results revealed several significant results. Perceived autonomy strongly predicted both autonomy support and motivation, confirming its central role in fostering AI-driven motivation. Relatedness and value also emerged as significant predictors of autonomy support, and competence and relatedness positively influenced autonomous motivation. Importantly, autonomy support enhanced both autonomous and student motivation, with the former strongly contributing to the latter. Student motivation was the most powerful driver of student engagement (*β* = 0.74, *p* < 0.001). Conversely, perceived expectancy showed no significant influence on either autonomy support or autonomous motivation, and the direct links from autonomy support and autonomous motivation to engagement were non-significant. These findings suggest that motivation operates as a critical mediator, translating psychological needs and AI-related perceptions into engagement outcomes. This study extends SDT by integrating AI-specific constructs and demonstrating how autonomy-supportive environments influence motivation pathways in digital learning contexts. This underscores that the value and relatedness associated with AI tools, along with perceived autonomy, are vital for sustaining motivation and engagement.

The results highlight the need for educators, developers, and policymakers to design AI-enabled learning environments that support autonomy, competence, and social connectedness while aligning tools with students’ values. This can enhance motivation, improve engagement, and ultimately lead to better learning outcomes. By emphasizing motivation as the key mediator, this study provides actionable insights into effective and sustainable AI integration in education.

### 7.1 Limitations and future research

Despite its theoretical and practical contributions, this study has several limitations that should be acknowledged. First, the study was conducted using data collected from a single higher education institution, King Saud University, with a sample of 297 students. Although the sample size was adequate for PLS-SEM analysis, the findings may not be fully generalizable to other universities, educational systems, or cultural contexts. Future studies should employ larger and more diverse samples across multiple institutions and countries to enhance the external validity of the findings.

Second, the study relied on self-reported survey data, which may be subject to response bias and social desirability effects. Although common method bias assessments indicated no serious concerns, future research could adopt mixed-methods approaches by combining surveys with interviews, focus groups, classroom observations, or learning analytics data to obtain a more comprehensive understanding of students’ experiences with Generative AI.

Third, the cross-sectional research design limits the ability to establish causal relationships among the investigated constructs. Longitudinal and experimental studies are therefore recommended to examine how students’ motivation, engagement, and perceptions of AI evolve over time and in response to different instructional interventions.

From a methodological perspective, future research may extend the present work by employing advanced analytical techniques, such as Artificial Neural Networks (ANN), Explainable Artificial Intelligence (XAI), and machine learning-based predictive models, to capture complex and non-linear relationships among variables. Furthermore, future studies should investigate additional mediating and moderating variables, including AI literacy, self-regulated learning, emotional engagement, teacher support, trust in AI, and institutional readiness.

Examining these factors would provide deeper insights into the mechanisms through which Generative AI influences student motivation, engagement, and learning outcomes in higher education.

## Data Availability

The original contributions presented in the study are included in the article/supplementary material, further inquiries can be directed to the corresponding author.

## References

[ref1] Abubakar S. Jeilani A. Yusuf M. (2025). The role of over-reliance on AI in the negative consequences of student learning: the moderating effects of ethical concerns and institutional policies. Cogent Educ. 12:2591503. doi: 10.1080/2331186X.2025.2591503

[ref2] Adekoya O. D. Ajonbadi H. A. Mordi C. Samuel P. Nguyen H. (2026). ‘A double‐edged tool’: a psychological needs perspective of generative AI and postgraduate international students' engagement in UK higher education. Br. Educ. Res. J. 1–19. doi: 10.1002/berj.70138

[ref3] Aithal P. S. Aithal S. (2023). Application of ChatGPT in higher education and research–a futuristic analysis. Int. J. Appl. Eng. Manag. Lett. 7, 168–194.

[ref4] Ala M. Shahid S. Mahmud S. Mohyuddin S. Kaur K. (2025). Perceived influence of GenAI on student engagement in online higher education. J. Appl. Learn. Teach. 8, 67–80. doi: 10.37074/jalt.2025.8.2.16

[ref5] Alamri H. Lowell V. Watson W. Watson S. L. (2020). Using personalized learning as an instructional approach to motivate learners in online higher education: learner self-determination and intrinsic motivation. J. Res. Technol. Educ. 52, 322–352. doi: 10.1080/15391523.2020.1728449

[ref6] Almogren A. S. Al-Rahmi W. M. Dahri N. A. (2024). Exploring factors influencing the acceptance of ChatGPT in higher education: A Smart education perspective. Heliyon 10:e31887. doi: 10.1016/j.heliyon.2024.e31887, 38845866 PMC11154614

[ref7] Al-Rahmi W. M. Dahri N. A. Dahri F. H. Aldaijy A. Alyoussef I. Y. “Investigating the Adoption of Ai-Based MOOCS as A Smart Digital Learning Environment: Using SEM Analysis Approach,” (2026). Preprint/Zenodo record.

[ref8] Al-Rahmi W. M. Yahaya N. Alamri M. M. Alyoussef I. Y. Al-Rahmi A. M. Kamin Y. B. (2021). Integrating innovation diffusion theory with technology acceptance model: supporting students’ attitude towards using a massive open online courses (MOOCs) systems. Interact. Learn. Environ. 29, 1380–1392. doi: 10.1080/10494820.2019.1629599

[ref9] Alwakid W. N. Dahri N. A. (2025a). Harnessing AI capabilities and green entrepreneurial orientation for sustainable SME performance using SEM analysis approach. Technol. Soc. 83:103007. doi: 10.1016/j.techsoc.2025.103007

[ref10] Alwakid W. N. Dahri N. A. (2025b). Adopting blockchain gamification in education: exploring security, transparency, and trust using SEM analysis. Educ. Inf. Technol. 31, 447–485. doi: 10.1007/s10639-025-13777-1

[ref11] Alwakid W. N. Dahri N. A. Humayun M. Alwakid G. N. (2025). Integrating AI chatbots for enhancing academic support in business education: a SEM-based study toward sustainable learning. Int. J. Manag. Educ. 23:101252. doi: 10.1016/j.ijme.2025.101252

[ref12] Azevedo R. Bouchet F. Duffy M. Harley J. Taub M. Trevors G. . (2022). Lessons learned and future directions of metatutor: leveraging multichannel data to scaffold self-regulated learning with an intelligent tutoring system. Front. Psychol. 13:813632. doi: 10.3389/fpsyg.2022.813632, 35774935 PMC9239319

[ref13] Bai X. Yang L. (2025). Research on the influencing factors of generative artificial intelligence usage intent in post-secondary education: an empirical analysis based on the AIDUA extended model. Front. Psychol. 16:1644209. doi: 10.3389/fpsyg.2025.1644209, 41040103 PMC12484213

[ref14] Batista J. Mesquita A. Carnaz G. (2024). Generative AI and higher education: trends, challenges, and future directions from a systematic literature review. Information 15:676. doi: 10.3390/info15110676

[ref15] Bilquise G. Ibrahim S. Salhieh S. M. (2023). Investigating student acceptance of an academic advising chatbot in higher education institutions. Educ. Inf. Technol. 29, 6357–6382. doi: 10.1007/s10639-023-12076-x

[ref16] Calvo R. A. Peters D. Vold K. Ryan R. M. (2020). “Supporting human autonomy in AI systems: a framework for ethical enquiry,” in Christopher Burr and Luciano Floridi (Eds.), Ethics of Digital Well-Being: A Multidisciplinary Approach, (Cham, Switzerland: Springer International Publishing), 31–54.

[ref17] Chiu T. K. F. (2021). Digital support for student engagement in blended learning based on self-determination theory. Comput. Hum. Behav. 124:106909. doi: 10.1016/j.chb.2021.106909

[ref18] Chiu T. K. F. Moorhouse B. L. Chai C. S. Ismailov M. (2023). Teacher support and student motivation to learn with artificial intelligence (AI) based chatbot. Interact. Learn. Environ. 32, 1–17. doi: 10.1080/10494820.2023.2172044

[ref19] Chugh P. Jain V. (2026). From adoption to augmentation: a TCCM analysis of generative AI in higher education. Qual. Quant. 1, 23–40. doi: 10.1007/s11135-026-02718-w

[ref20] Cohen J. (1988). Statistical Power Analysis for the Behavioral Sciences. New York, NY: Academic Press.

[ref21] Cui Y. van Esch P. (2022). Autonomy and control: how political ideology shapes the use of artificial intelligence. Psychol. Mark. 39, 1218–1229. doi: 10.1002/mar.21643

[ref22] Dahri N. A. Al-Rahmi W. M. Almogren A. S. Yahaya N. Vighio M. S. Al-Maatuok Q. (2023a). Mobile-based training and certification framework for teachers’ professional development. Sustainability 15:5839. doi: 10.3390/su15075839

[ref23] Dahri N. A. Al-Rahmi W. M. Almogren A. S. Yahaya N. Vighio M. S. Al-maatuok Q. . (2023b). Acceptance of mobile learning technology by teachers: influencing mobile self-efficacy and 21st-century skills-based training. Sustainability 15:8514. doi: 10.3390/su15118514

[ref24] Dahri N. A. Dahri F. H. Laghari A. A. Javed M. (2025). Decoding ChatGPT’s impact on student satisfaction and performance: a multimodal machine learning and explainable AI approach. Complex Eng. Syst. 5:N-A. doi: 10.20517/ces.2025.07

[ref25] Dahri N. A. Moskalenko N. (2024). Emotions and cognitive processes in labor activity: the role of emotional intelligence. Qainar J. Soc. Sci. 3, 24–38. doi: 10.58732/2958-7212-2024-3-24-38

[ref26] Dahri N. A. Vighio M. S. Das Bather J. Arain A. A. (2021). Factors influencing the acceptance of mobile collaborative learning for the continuous professional development of teachers. Sustainability 13:13222. doi: 10.3390/su132313222

[ref27] Dahri N. A. Yahaya N. al-Rahmi W. M. Aldraiweesh A. Alturki U. Almutairy S. . (2024a). Extended TAM based acceptance of AI-powered ChatGPT for supporting metacognitive self-regulated learning in education: A mixed-methods study. Heliyon 10:e29317. doi: 10.1016/j.heliyon.2024.e29317, 38628736 PMC11016976

[ref28] Dahri N. A. Yahaya N. Al-Rahmi W. M. Almogren A. S. Vighio M. S. (2024b). Investigating factors affecting teachers’ training through mobile learning: task technology fit perspective. Educ. Inf. Technol. 29, 1–37. doi: 10.1007/s10639-023-12434-9

[ref29] Dahri N. A. Yahaya N. Al-Rahmi W. M. Noman H. A. Alblehai F. Kamin Y. B. . (2024c). Investigating the motivating factors that influence the adoption of blended learning for teachers’ professional development. Heliyon 10:e34900. doi: 10.1016/j.heliyon.2024.e34900, 39145035 PMC11320316

[ref30] Dahri N. A. Yahaya N. al-Rahmi W. M. Vighio M. S. Alblehai F. Soomro R. B. . (2024d). Investigating AI-based academic support acceptance and its impact on students’ performance in Malaysian and Pakistani higher education institutions. Educ. Inf. Technol. 29, 18695–18744. doi: 10.1007/s10639-024-12599-x, 30311153

[ref31] Davis F. D. (1989). Perceived usefulness, perceived ease of use, and user acceptance of information technology. MIS Q. 13, 319–340. doi: 10.2307/249008

[ref32] Davis F. D. Bagozzi R. P. Warshaw P. R. (1989). User acceptance of computer technology: A comparison of two theoretical models. Manag. Sci. 35, 982–1003. doi: 10.1287/mnsc.35.8.982, 19642375

[ref33] Deci E. L. Eghrari H. Patrick B. C. Leone D. R. (1994). Facilitating internalization: the self-determination theory perspective. J. Pers. 62, 119–142. doi: 10.1111/j.1467-6494.1994.tb00797.x, 8169757

[ref34] Eccles J. S. Wigfield A. (2023). Expectancy-value theory to situated expectancy-value theory: reflections on the legacy of 40+ years of working together. Motiv. Sci. 9:1. doi: 10.1037/mot0000288

[ref35] Eccles J. S. Wigfield A. (2024). The development, testing, and refinement of Eccles, Wigfield, and colleagues’ situated expectancy-value model of achievement performance and choice. Educ. Psychol. Rev. 36, 1–29. doi: 10.1007/s10648-024-09888-9, 30311153

[ref36] Etikan I. Musa S. A. Alkassim R. S. (2016). Comparison of convenience sampling and purposive sampling. Am. J. Theor. Appl. Stat. 5, 1–4. doi: 10.11648/j.ajtas.20160501.11

[ref37] Fornell C. Larcker D. F. (1981). Structural Equation Models with Unobservable Variables and Measurement Error: Algebra and Statistics. Los Angeles: Sage Publications.

[ref38] Gunter G. A. Reeves J. L. (2017). Online professional development embedded with mobile learning: an examination of teachers’ attitudes, engagement and dispositions. Br. J. Educ. Technol. 48, 1305–1317. doi: 10.1111/bjet.12490

[ref39] Haerens L. Vansteenkiste M. De Meester A. Delrue J. Tallir I. Vande Broek G. . (2018). Different combinations of perceived autonomy support and control: identifying the most optimal motivating style. Phys. Educ. Sport Pedagog. 23, 16–36. doi: 10.1080/17408989.2017.1346070

[ref40] Hair J. Alamer A. (2022). Partial least squares structural equation modeling (PLS-SEM) in second language and education research: guidelines using an applied example. Res. Methods Appl. Linguist. 1:100027. doi: 10.1016/j.rmal.2022.100027

[ref41] Hair J. F. Black W. C. Babin B. Anderson R. E. (2010). Multivariate Data Analysis: A Global Perspective. New Jersey: Pearson Prentice Hall.

[ref42] Hair J. F. Jr. Sarstedt M. Hopkins L. Kuppelwieser V. G. (2014). Partial least squares structural equation modeling (PLS-SEM) an emerging tool in business research. Eur. Bus. Rev. 26, 106–121. doi: 10.1108/EBR-10-2013-0128

[ref43] Hair J. F. Risher J. J. Sarstedt M. Ringle C. M. (2019). When to use and how to report the results of PLS-SEM. Eur. Bus. Rev. 31, 2–24. doi: 10.1108/ebr-11-2018-0203

[ref44] Henseler J. Ringle C. M. Sarstedt M. (2015). A new criterion for assessing discriminant validity in variance-based structural equation modeling. J. Acad. Mark. Sci. 43, 115–135. doi: 10.1007/s11747-014-0403-8

[ref45] Hidayat-ur-Rehman I. (2024). Examining AI competence, chatbot use and perceived autonomy as drivers of students’ engagement in informal digital learning. J. Res. Innov. Teach. Learn. 17, 196–212. doi: 10.1108/jrit-05-2024-0136

[ref46] Hooda M. Rana C. Dahiya O. Rizwan A. Hossain M. S. (2022). Artificial intelligence for assessment and feedback to enhance student success in higher education. Math. Probl. Eng. 2022:5215722. doi: 10.1155/2022/5215722

[ref47] Hu Q. Lu Y. Pan Z. Gong Y. Yang Z. (2021). Can AI artifacts influence human cognition? The effects of artificial autonomy in intelligent personal assistants. Int. J. Inf. Manag. 56:102250. doi: 10.1016/j.ijinfomgt.2020.102250, 38826717

[ref48] Huang J. Tan M. (2023). The role of ChatGPT in scientific communication: writing better scientific review articles. Am. J. Cancer Res. 13:1148. 37168339 PMC10164801

[ref49] Ifinedo P. (2017). Examining students’ intention to continue using blogs for learning: perspectives from technology acceptance, motivational, and social-cognitive frameworks. Comput. Hum. Behav. 72, 189–199. doi: 10.1016/j.chb.2016.12.049

[ref50] Jeno L. M. Nylehn J. Hole T. N. Raaheim A. Velle G. Vandvik V. (2023). Motivational determinants of students’ academic functioning: the role of autonomy-support, autonomous motivation, and perceived competence. Scand. J. Educ. Res. 67, 194–211. doi: 10.1080/00313831.2021.1990125

[ref51] Jo H. (2023). Understanding AI tool engagement: a study of ChatGPT usage and word-of-mouth among university students and office workers. Telemat. Inform. 85:102067. doi: 10.1016/j.tele.2023.102067

[ref52] Kock N. (2016). Hypothesis testing with confidence intervals and P values in PLS-SEM. Int. J. e-Collab. 12, 1–6. doi: 10.4018/ijec.2016070101

[ref53] Lalicic L. Weismayer C. (2021). Consumers’ reasons and perceived value co-creation of using artificial intelligence-enabled travel service agents. J. Bus. Res. 129, 891–901. doi: 10.1016/j.jbusres.2020.11.005

[ref54] Lazorak O. Belkina O. Yaroslavova E. (2021). Changes in student autonomy via e-learning courses. Int. J. Emerg. Technol. Learn. 16, 209–225. doi: 10.3991/ijet.v16i17.23863

[ref55] Lemos A. Wulf G. Lewthwaite R. Chiviacowsky S. (2017). Autonomy support enhances performance expectancies, positive affect, and motor learning. Psychol. Sport Exerc. 31, 28–34. doi: 10.1016/j.psychsport.2017.03.009

[ref56] Lin S.-H. Huang Y.-C. (2018). Assessing college student engagement: development and validation of the student course engagement scale. J. Psychoeduc. Assess. 36, 694–708. doi: 10.1177/0734282917697618

[ref57] Luo Y. Day M. J. (2026). Determinants of lecturer readiness to adopt generative AI in higher education: survey evidence from UTAUT and self-determination theory. Educ. Inf. Technol. 31, 3399–3430. doi: 10.1007/s10639-026-13931-3

[ref58] Luo Y. Lin J. Yang Y. (2021). Students’ motivation and continued intention with online self-regulated learning: A self-determination theory perspective. Z. Erziehwiss. 24, 1379–1399. doi: 10.1007/s11618-021-01042-3, 34483723 PMC8404548

[ref59] Madjar N. Assor A. (2013). “Two types of perceived control over learning: perceived efficacy and perceived autonomy,” in John Hattie and Eric M. Anderman (Eds.), International Guide to Student Achievement, (New York, NY: Routledge), 439–441.

[ref60] Makri M. Gkioka M. Moraitou D. Fidani L. Tegos T. Tsolaki M. (2023). Attitudes, motivations, and barriers to pre-symptomatic Alzheimer’s disease screening: Development and validation of the PRE-ADS questionnaire. Journal of Alzheimer’s Disease, 95, 273–286. doi: 10.3233/JAD-230214 37661876

[ref61] Malinka K. Peresíni M. Firc A. Hujnák O. Janus F. On the educational impact of chatgpt: is artificial intelligence ready to obtain a university degree? Proceedings of the 2023 Conference on Innovation and Technology in Computer Science Education V. 1. New York, NY, USA: ACM. (2023) 47–53.

[ref62] Manning P. J. (2012). Use your words: providing informational feedback as a means to support self-determination and improve law student outlook and outcomes. Cumb. L. Rev. 43:225. doi: 10.2139/ssrn.1967280

[ref63] Ng J. W. Naval M. A. (2025). Technological acceptance and motivation of generative artificial intelligence (GenAI) in sport education 2025 2nd International Conference on Artificial Intelligence and Teacher Education, ICAITE 2025. Piscataway, NJ, USA: IEEE. 304–308.

[ref64] Niemiec C. P. Ryan R. M. Deci E. L. (2009). The path taken: consequences of attaining intrinsic and extrinsic aspirations in post-college life. J. Res. Pers. 43, 291–306. doi: 10.1016/j.jrp.2008.09.001, 20161160 PMC2736104

[ref65] Nunnally B. Bernstein I. (1994). Psychometric Theory. New York: Oxford University Press.

[ref66] Ode E. Nana R. Boro I. O. Ikyanyon D. N. (2025). A cross-country analysis of self-determination and continuance use intention of AI tools in business education: does instructor support matter? Comput. Educ. Artif. Intell. 8:100402. doi: 10.1016/j.caeai.2025.100402

[ref67] Otman S. B. Otman S. B. Adalı G. K. (2025). The impact of generative AI on university students’ learning experience: a study on cognitive and affective outcomes. J. Inf. Organ. Sci. 49, 329–344. doi: 10.31341/jios.49.2.10

[ref68] Parrisius C. K. (2020). The Unfolding of Students’ Motivation in the Natural Classroom Setting: The Role of Motivational Teaching Practices. Tübingen, Germany: Universität Tübingen.

[ref69] Pasupuleti R. S. Jangam D. C. Bhimavarapu A. Gunnam V. R. Sikhakolli V. R. Thiyyagura D. (2025). The role of learning motivation factors in Deepseek generative AI adoption among higher education students in India. Electron. J. e-Learn. 23, 1–14. doi: 10.34190/ejel.23.4.4245

[ref70] Petrick J. F. (2002). Development of a multi-dimensional scale for measuring the perceived value of a service. J. Leis. Res. 34, 119–134. doi: 10.1080/00222216.2002.11949965

[ref72] Pintrich P. R. (2003). A motivational science perspective on the role of student motivation in learning and teaching contexts. J. Educ. Psychol. 95, 667–686. doi: 10.1037/0022-0663.95.4.667

[ref73] Podsakoff P. M. MacKenzie S. B. Lee J.-Y. Podsakoff N. P. (2003). Common method biases in behavioral research: a critical review of the literature and recommended remedies. J. Appl. Psychol. 88, 879–903. doi: 10.1037/0021-9010.88.5.879, 14516251

[ref74] Prentzas J. Sidiropoulou M. (2023). Assessing the use of open AI chat-GPT in a University Department of Education 2023 14th International Conference on Information, Intelligence, Systems & Applications (IISA). Piscataway, NJ, USA: IEEE 1–4.

[ref75] Prihadi K. Hairul N. I. Hazri J. (2012). Mediation effect of locus of control on the causal relationship between students’ perceived teachers’ expectancy and self-esteem. Electron. J. Res. Educ. Psychol. 10, 713–736.

[ref76] Reeve J. (2009). Why teachers adopt a controlling motivating style toward students and how they can become more autonomy supportive. Educ. Psychol. 44, 159–175. doi: 10.1080/00461520903028990

[ref77] Ryan R. M. (1991). “The nature of the self in autonomy and relatedness,” in Jetse M. M. Suls & Anthony G. Greenwald (Eds.), The Self: Interdisciplinary Approaches, (Hillsdale, NJ: Lawrence Erlbaum Associates), 208–238.

[ref78] Ryan R. M. Deci E. L. (2008). From ego depletion to vitality: theory and findings concerning the facilitation of energy available to the self. Soc. Personal. Psychol. Compass 2, 702–717. doi: 10.1111/j.1751-9004.2008.00098.x

[ref79] Ryan R. M. Deci E. L. (2017). Self-Determination Theory: Basic Psychological Needs in Motivation, Development, and Wellness, vol. 10 Guilford Press, 28806.

[ref80] Ryan R. M. Deci E. L. (2020). Intrinsic and extrinsic motivation from a self-determination theory perspective: definitions, theory, practices, and future directions. Contemp. Educ. Psychol. 61:101860. doi: 10.1016/j.cedpsych.2020.101860

[ref81] Şahin F. Doğan E. İlic U. (2024). Instructors’ Continuance Intention to Use Technology in Online and Hybrid Settings: Integrating Psychological Needs and Emotions. International Journal of Human–Computer Interaction, 41, 1028–1041. doi: 10.1080/10447318.2024.2309002

[ref82] Sánchez-Fernández R. Iniesta-Bonillo M. Á. (2007). The concept of perceived value: a systematic review of the research. Mark. Theory 7, 427–451. doi: 10.1177/1470593107083165

[ref83] Shoukat R. (2024). Harnessing AI for educational transformation: a comparative study of China, India and Pakistan. Strateg. Stud. 44, 89–108. doi: 10.53532/ss.044.01.00352

[ref84] Skinner E. A. Belmont M. J. (1993). Motivation in the classroom: reciprocal effects of teacher behavior and student engagement across the school year. J. Educ. Psychol. 85, 571–581. doi: 10.1037/0022-0663.85.4.571

[ref85] Skinner E. A. Pitzer J. R. Steele J. S. (2016). Can student engagement serve as a motivational resource for academic coping, persistence, and learning during late elementary and early middle school? Dev. Psychol. 52, 2099–2117. doi: 10.1037/dev0000232, 27893248

[ref86] Raffaghelli J. E. Rodríguez M. E. Guerrero-Roldán A.-E. Bañeres D. (2012). Applying the UTAUT model to explain the students’ acceptance of an early warning system in higher education. Sustainability 13, 486–490. doi: 10.3390/su13041946

[ref87] Song C. Song Y. (2023). Enhancing academic writing skills and motivation: assessing the efficacy of ChatGPT in AI-assisted language learning for EFL students. Front. Psychol. 14:1260843. doi: 10.3389/fpsyg.2023.1260843, 38162975 PMC10754989

[ref88] Soomro R. B. Memon S. G. Dahri N. A. Al-Rahmi W. M. Aldriwish K. Salameh A. . (2024). The adoption of digital technologies by small and medium-sized enterprises for sustainability and value creation in Pakistan: the application of a two-staged hybrid SEM-ANN approach. Sustainability 16:7351. doi: 10.3390/su16177351

[ref89] Srinivasa K. G. Kurni M. Saritha K. (2022). “Harnessing the power of AI to education,” in Learning, Teaching, and Assessment Methods for Contemporary Learners: Pedagogy for the Digital Generation, (Singapore: Springer Nature Singapore), 311–342.

[ref90] Tannady H. Dewi C. S. (2024). Exploring role of technology performance expectancy, application effort expectancy, perceived risk and perceived cost on digital behavioral intention of GoFood users. J. Inf. Teknol. 6, 80–85. doi: 10.60083/jidt.v6i1.477

[ref91] Tao Y. Meng Y. Gao Z. Yang X. (2022). Perceived teacher support, student engagement, and academic achievement: a meta-analysis. Educ. Psychol. 42, 401–420. doi: 10.1080/01443410.2022.2033168

[ref92] Tbaishat D. AlFandi O. Hamad F. Bukhari S. M. S. Al Muhaissen S. (2026). Modeling generative AI adoption in higher education: an integrated TAM–TPB–SDT framework with SEM validation. Comput. Educ. Artif. Intell. 10:100541. doi: 10.1016/j.caeai.2026.100541

[ref93] van der Vorst T. Jelicic N. “Artificial Intelligence in Education: Can AI Bring the full Potential of Personalized Learning to Education?”. Calgary, Canada: International Telecommunications Society (ITS). (2019).

[ref94] Vansteenkiste M. Lens W. Deci E. L. (2006). Intrinsic versus extrinsic goal contents in self-determination theory: another look at the quality of academic motivation. Educ. Psychol. 41, 19–31. doi: 10.1207/s15326985ep4101_4

[ref95] Vashishth T. K. Sharma V. Sharma K. K. Kumar B. Panwar R. Chaudhary S. (2024). “AI-driven learning analytics for personalized feedback and assessment in higher education,” in Tien V. T. Nguyen (Ed.), Using Traditional Design Methods to Enhance AI-Driven Decision Making, (Hershey, PA: IGI Global), 206–230.

[ref96] Vaughan N. (2014). Student engagement and blended learning: making the assessment connection. Educ. Sci. 4, 247–264. doi: 10.3390/educsci4040247

[ref97] Venkatesh V. Davis F. D. (2000). A theoretical extension of the technology acceptance model: four longitudinal field studies. Manag. Sci. 46, 186–204. doi: 10.1287/mnsc.46.2.186.11926

[ref98] Wei L. (2023). Artificial intelligence in language instruction: impact on English learning achievement, L2 motivation, and self-regulated learning. Front. Psychol. 14:1261955. doi: 10.3389/fpsyg.2023.1261955, 38023040 PMC10658009

[ref99] Wigfield A. Eccles J. S. (2000). Expectancy–value theory of achievement motivation. Contemp. Educ. Psychol. 25, 68–81. doi: 10.1006/ceps.1999.1015, 10620382

[ref100] Wood D. R. (2016). The Impact of Students’ Perceived Relatedness and Competence upon their Motivated Engagement with Learning Activities: A Self-Determination Theory Perspective. Birmingham, UK: University of Birmingham.

[ref102] Xia Q. Chiu T. K. F. Lee M. Sanusi I. T. Dai Y. Chai C. S. (2022). A self-determination theory (SDT) design approach for inclusive and diverse artificial intelligence (AI) education. Comput. Educ. 189:104582. doi: 10.1016/j.compedu.2022.104582

[ref103] Yang Y. Wen X. Maidin S. S. Generative AI tools in higher education emerging research: A bibliometric analysis of co-citation and co-word analysis Proceedings of 2024 3rd International Conference on Artificial Intelligence and Education, ICAIE 2024. New York, NY, USA: ACM. (2025) 166–174

[ref104] Yilmaz R. Yilmaz F. G. K. (2023). The effect of generative artificial intelligence (AI)-based tool use on students’ computational thinking skills, programming self-efficacy and motivation. Comput. Educ. Artif. Intell. 4:100147. doi: 10.1016/j.caeai.2023.100147

[ref105] Yuce A. Abubakar A. M. Ilkan M. (2019). Intelligent tutoring systems and learning performance: applying task-technology fit and IS success model. Online Inf. Rev. 43, 600–616. doi: 10.1108/OIR-11-2017-0340

[ref106] Yurt E. Kasarci I. (2024). A questionnaire of artificial intelligence use motives: a contribution to investigating the connection between AI and motivation. Int. J. Technol. Educ. 7, 308–325. doi: 10.46328/ijte.750

[ref107] Zepke N. (2011). Understanding teaching, motivation and external influences in student engagement: how can complexity thinking help? Res. Post-Compuls. Educ. 16, 1–13. doi: 10.1080/13596748.2011.549721

[ref108] Zhou L. Li J. J. (2023). The impact of ChatGPT on learning motivation: a study based on self-determination theory. Educ. Sci. Manag. 1, 19–29. doi: 10.56578/esm010103

